# Advanced Nb-Based MOF-Supported Co/Pt Nanocatalyst for Sustainable Hydrogen Production from Sodium Borohydride

**DOI:** 10.1021/acsomega.6c01980

**Published:** 2026-05-18

**Authors:** Giovana Barros Magalhães, Tatianny de Araujo Andrade, Renata Lopes Moreira, Renê Chagas da Silva, Gilberto Rodrigues da Silva Junior, Jemmyson Romário de Jesus

**Affiliations:** † Research Laboratory in bionanomaterials, LPbio, Department of Chemistry, Federal University of Viçosa, Viçosa, Minas Gerais 36570-900, Brazil; ‡ Department of Chemistry, 28120 Federal University of Viçosa, Viçosa, Minas Gerais 36570-900, Brazil; § Department of Physics, 28120 Federal University of Viçosa, Viçosa, Minas Gerais 36570-900, Brazil; ∥ National Institute of Science and Technology of Bioanalytics-Lauro Kubota (INCTBio-LK), Instituto de Química, Universidade Estadual de Campinas, Campinas, São Paulo 13083970, Brazil

## Abstract

Expanding renewable energy sources is essential for global decarbonization. Hydrogen (H_2_) is a clean energy carrier, but its storage remains challenging. Sodium borohydride (NaBH_4_) is attractive due to its high hydrogen content; however, its hydrolysis is kinetically slow and requires catalysts. In this study, a niobium-based Metal–Organic Framework, Nb­(PDC)­(BTC), where PDC is pyridine-2,5-dicarboxylate and BTC is benzene-1,3,5-tricarboxylate, was synthesized and used as a support for cobalt–platinum nanoparticles (Co–Pt NP) formed in situ. Fourier transform infrared spectroscopy (FT-IR) confirmed ligand coordination, X-ray diffraction (XRD) demonstrated the crystalline phase, and thermogravimetric analysis (TGA) showed good thermal stability. The Brunauer–Emmett–Teller (BET) surface area reached 1839.63 m^2^/g. Scanning electron microscopy (SEM) and high-resolution transmission electron microscopy (HRTEM) revealed rough crystalline morphology and well-dispersed spherical nanoparticles anchored to the support. Energy-dispersive X-ray spectroscopy (EDS) verified the presence of Nb, Co, and Pt. The catalyst achieved a hydrogen generation rate (HGR) of 3214 mL min^–1^ g^–1^ under optimal conditions (0.5 mmol of Co_60_Pt_40_ on 10 mg of Nb­(PDC)­(BTC); NaBH_4_ 0.500 mol L^–1^ in sodium hydroxide (NaOH) 0.100 mol L^–1^ at 301.15 K), with an activation energy of 17.45 kJ mol^–1^, demonstrating strong potential for sustainable H_2_ production.

## Graphical abstract

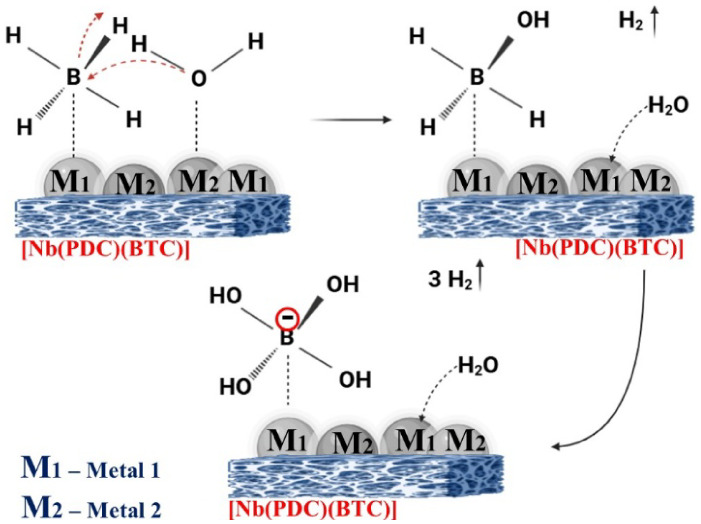

## 1 Introduction

One of the most important global challenges today is decarbonizing the world economy. Expanding renewable energy sources is widely regarded as a key strategy to address this problem by promoting sector integration. This approach involves blending renewable energy into industrial, energy, and transportation systems through innovative, clean-energy solutions.^1^ In this context, hydrogen (H_2_) emerges as a promising alternative for mitigating the environmental impacts associated with greenhouse gas emissions, such as carbon dioxide (CO_2_), nitrogen oxides (NO_*x*_), and methane (CH_4_), which significantly contribute to global warming.^2^ The H_2_ is considered an attractive energy carrier due to its potential for clean and sustainable energy storage and release. Its production is primarily aimed at reducing carbon emissions by substituting fossil fuels such as coal, oil, and natural gas, thereby playing a key role in advancing the global energy transition.^3^

Despite the growing interest in H_2_ production, several challenges remain, particularly those associated with transportation, storage, cost, infrastructure, and gas purity.^4^ Among the different strategies for solid-state H_2_ storage, metal hydrides, such as sodium borohydride (NaBH_4_), have shown considerable promise. The H_2_ release from NaBH_4_ occurs spontaneously via hydrolysis; however, the reaction is characterized by slow kinetics, thereby necessitating the use of catalysts to enhance the efficiency and rate of H_2_ generation.^5^ Metal catalysts such as platinum (Pt), palladium (Pd), nickel (Ni), and cobalt (Co) have garnered considerable attention due to their outstanding catalytic efficiency in reducing activation energy and enhancing the kinetics of the H_2_ evolution reaction.^6,7^ Furthermore, immobilizing these nanocatalysts on solid substrates enhances the dispersion and stability of the catalytic system by preventing the aggregation and detachment of nanoparticles. This strategy ensures the durability of the active sites and sustains catalytic performance in H_2_ generation.^8^

Considering these factors, the search for robust and versatile support materials has intensified, with metal–organic frameworks (MOFs) emerging as promising candidates for catalytic applications.^5,9^ These hybrid materials, composed of metal ions or clusters coordinated to organic linkers, form highly ordered three-dimensional networks with tunable properties. MOFs stand out for their high surface area, well-defined porosity, structural crystallinity, chemical and thermal stability, and reusability. Their abundance of accessible active sites further reinforces their suitability for use in heterogeneous catalysis.^10,11^

Given the above, this study presents the synthesis and application of a niobium (Nb)-based MOF functionalized with Pt and Co nanoparticles for H_2_ generation via NaBH_4_ hydrolysis. The selection of Nb is particularly relevant, as Brazil holds some of the largest global reserves of this element, whose catalytic potential remains little explored.^12^ Thus, the advancement of Nb-based MOFs offers a promising route to enhance the technical, economic, and environmental feasibility of sustainable H_2_ production, contributing to decarbonization pathways and supporting the development of strategic energy materials at the national level.

## 2 Materials and Methods

### 2.1 Standards and Reagents

All reagents used in this study were of analytical grade. The 2,5-pyridinedicarboxylic acid (PDC), 1.5 g of 1,3,5-benzenetricarboxylic acid (BTC), and potassium hexachloroplatinate (IV) (K_2_PtCl_6_ 38–41%) were purchased from Sigma-Aldrich (St. Louis, MO, USA). Ammonium niobium oxalate (AmNbOx) was sourced from CBMM (Minas Gerais, Brazil). Sodium borohydride (NaBH_4_ 98%), nickel sulfate heptahydrate (NiSO_4_·7H_2_O 98%), cobalt­(II) nitrate hexahydrate (Co­(NO_3_)_2_·6H_2_O, 99.9%), and sodium hydroxide (NaOH) were obtained from VETEC (Rio de Janeiro, Brazil). All solutions were prepared using Type 1 water produced by a Milli-Q purification system (Millipore Corporation).

### 2.2 Synthesis of the Nb-Based Coordination Compound ([Nb­(PDC)­(BTC)])

The [Nb­(PDC)­(BTC)] was synthesized via a solvothermal method, based on the procedure reported by Jesus et al.,^13^ with modifications. Initially, 0.50 g of AmNbOx was dissolved in 10.00 mL of Type 1 water under stirring. Subsequently, 0.75 g of BTC and 0.75 g of PDC were dissolved in 10.00 mL of Type 1 water. The pH of this ligand solution was adjusted to approximately 8.0 using NaOH (8 mol L^–1^), and it was then added to the AmNbOx solution. The resulting mixture was stirred for 40 min at room temperature.

The resulting solution was transferred to a reactor, to which 4.00 mL of ethylene glycol was added. The reactor was then placed in a stainless steel autoclave and heated to 200 °C for 24 h. After the reaction was completed, the autoclave was cooled to room temperature. The solid formed was collected by centrifugation at 4000 rpm for 15 min, followed by three successive washes with 2.00 mL of a 60% (v/v) ethanol solution to remove residual byproducts and unreacted ethylene glycol. The purified material was then dried in an oven at 50 °C for 8 h.

### 2.3 Synthesis of Metal Nanoparticles Decorated on [Nb­(PDC)­(BTC)]

The [Nb­(PDC)­(BTC)]/Co/Pt-NPs catalyst was synthesized following the Sperandio et al.^5^ procedure, with slight modifications. Briefly, 10 mg of the [Nb­(PDC)­(BTC)] support was dispersed in 10.00 mL of ultrapure water in a 25.00 mL beaker and stirred for 10 min to ensure uniform dispersion. Subsequently, platinum (K_2_PtCl_6_) and cobalt (Co­(NO_3_)_2_·6H_2_O) precursor salts were added, exploring the following Co/Pt molar ratios: 100:0, 80:20, 60:40, 40:60, 20:80, and 0:100. To reduce the metal ions, 1.00 mL of NaBH_4_ solution (1.05 mol L^–1^) was introduced, and the mixture was stirred for an additional 10 min. The suspension was then centrifuged (4000 rpm) for 10 min. The supernatant was discarded, and the resulting solid was washed with ultrapure water and centrifuged again under the same conditions. The resulting [Nb­(PDC)­(BTC)]/Co/Pt-NPs catalyst was characterized and subsequently utilized in the NaBH_4_ hydrolysis reaction for H_2_ generation.

### 2.4 Characterization of Materials

The material underwent characterization using several analytical techniques. Fourier-transform infrared spectroscopy (FT-IR) was employed to elucidate the functional groups present in the Nb-based coordination compound, [Nb­(PDC)­(BTC)]. Spectral data were acquired using a Bruker ALPHA II spectrometer equipped with an ATR accessory. Scans were conducted over the 400–4000 cm^–1^ range, with 54 coadded scans and a spectral resolution of 4 cm^–1^.

Thermogravimetric analysis (TGA) was conducted using a PerkinElmer analyzer (USA). Samples were heated from 30 to 900 °C at a constant rate of 10 °C min^–1^ under a nitrogen flow of 50 mL min^–1^. This dynamic setup continuously recorded mass loss, providing insights into phase changes and degradation stages. The diffraction pattern was obtained from powder X-ray diffraction (XRD) at room temperature using a Rigaku diffractometer in continuous Bragg–Brentano geometry, with Cu Kα radiation over a 2θ range of 25° to 70°.

Nitrogen adsorption–desorption isotherms for [Nb­(PDC)­(BTC)] were obtained using an Anton Paar Nova 600 Series instrument. Before analysis, the sample was vacuum-dried at 150 °C for 4 h to remove residual moisture and volatile gases. The specific surface area was calculated by the Brunauer–Emmett–Teller (BET) method, while the pore size distribution and volume were determined using the Barrett–Joyner–Halenda (BJH) model.

The morphology and elemental distribution of the [Nb­(PDC)­(BTC)] and [Nb­(PDC)­(BTC)]/Co/Pt-NPs were investigated by Scanning Electron Microscopy (SEM) and High-Resolution Transmission Electron Microscopy (HRTEM), both coupled with Energy Dispersive X-ray Spectrometer (EDS). SEM imaging was made using a JEOL (JSM-6010LA, Akishima, Tokyo, Japan), fitted with an Everhart–Thornley detector. Imaging was conducted at 20 kV acceleration voltage, with 4 nm resolution and magnifications ranging from 8× to 300,000×. HRTEM analysis employed a Tecnai G2–20—FEI SuperTwin microscope operated at 200 kV. Elemental mapping was carried out using an EDS equipped with a silicon drift detector, providing an energy resolution of approximately 133 eV.

### 2.5 Hydrolysis of NaBH_4_ for H_2_ Evolution

The H_2_ evolution experiment was performed following the procedure described by Souza et al.^8^ The catalyst [Nb­(PDC)­(BTC)]/Co/Pt-NPs were dispersed in 5.00 mL of ultrapure water and transferred to a Schlenk tube, which was then hermetically sealed. The tube outlet was connected to a buret, which in turn was connected to a water reservoir for leveling and pressure adjustment. After the system was assembled, 1.00 mL of a 0.500 mol L^–1^ NaBH_4_ solution was injected through a septum and the suspension was continuously stirred at a controlled temperature. As H_2_ was generated, it displaced water in the buret, allowing continuous monitoring of the reaction progress. The total H_2_ generated was quantified from the volume of water displaced over time.

#### 2.5.1 Optimization of Parameters for H_2_ Evolution

For the H_2_ evolution experiment from NaBH_4_ hydrolysis, various parameters influencing the reaction were optimized, including (i) the composition of bimetallic catalysts, (ii) the NaBH_4_ concentration, (iii) the NaOH concentration, and (iv) the temperature variation.

##### 2.5.1.1 Evaluation of Bimetallic Composition

The effect of the bimetallic composition of Co/Pt-NPs was evaluated at the following Co:Pt ratios (in mmol %): 100:0, 80:20, 60:40, 40:60, 20:80, and 0:100. All other experimental conditions stayed the same, including a temperature of 301.15 K, a support mass of 10 mg, and 1.00 mL of NaBH_4_ solution at 0.500 mol L^–1^.

##### 2.5.1.2 Evaluation of NaBH_4_ Concentration

The effect of NaBH_4_ concentration was assessed at 0.50, 0.75, 1.00, and 1.50 mol L^–1^. All other experimental parameters were kept constant: temperature (301.15 K), catalyst (0.5 mmol of Co_60_/Pt_40_ NPs supported on 10 mg of [Nb­(PDC)­(BTC)]), and a consistent volume of NaBH_4_ solution (1.00 mL) for each concentration tested.

##### 2.5.1.3 Evaluation of NaOH Concentration

The influence of NaOH was assessed using four different concentrations: 0.01, 0.05, 0.10, and 0.20 mol L^–1^. In each experiment, 0.500 mmol of NaBH_4_ was dissolved in 1.00 mL of NaOH at the specified concentration and then injected into the system. All other conditions were kept constant: temperature (301.15 K), and catalyst composition (0.5 mmol % Co_60_/Pt_40_-NPs and 10 mg support [Nb­(PDC)­(BTC)]).

##### 2.5.1.4 Evaluation of Temperature Effect

Different temperatures were evaluated (288.15, 301.15, 308.15, 318.15, and 328.15 K), with all other parameters kept constant, including catalyst (0.5 mmol % Co_60_/Pt_40_-NPs and 10 mg support [Nb­(PDC)­(BTC)]), and the use of 1.00 mL of NaBH_4_ solution at 0.500 mol L^–1^.

The activation energy (E_a_) was calculated using the Arrhenius equation (eq 1):

1 $$\ln\left(k\right)=\ln\left(A\right)-E_{a}/\mathrm{RT}$$

Where k represents the reaction rate constant, A denotes the pre-exponential factor, E_a_ is the apparent activation energy expressed in kJ mol^-–1^, R corresponds to the universal gas constant, and T refers to the absolute temperature.

## 3 Results and Discussion

### 3.1 Characterization of Materials

The FTIR spectra of the BTC and PDC ligands, as well as of the [Nb­(PDC)­(BTC)] compound, are shown in Figure 1A. The ligands displayed characteristic bands between 1029 and 1600 cm^–1^, which are associated with the stretching vibrations of C–O and C–C bonds in the aromatic rings. In the spectrum of [Nb­(PDC)­(BTC)], noticeable shifts in these bands were observed, confirming the successful coordination between Nb and the BTC and PDC ligands. Furthermore, the prominent shifts and absorption bands in the 500–920 cm^–1^ range can be attributed to the stretching and bending modes of Nb–O bonds, supporting the formation of coordinated interactions between the metal center and the carboxylate groups of the organic linkers.^14^ These findings are consistent with those reported by Sperandio et al., who observed similar results during the synthesis of a niobium and BTC-based nanocomposite inspired by MOF structures.^5^

**Figure 1:**
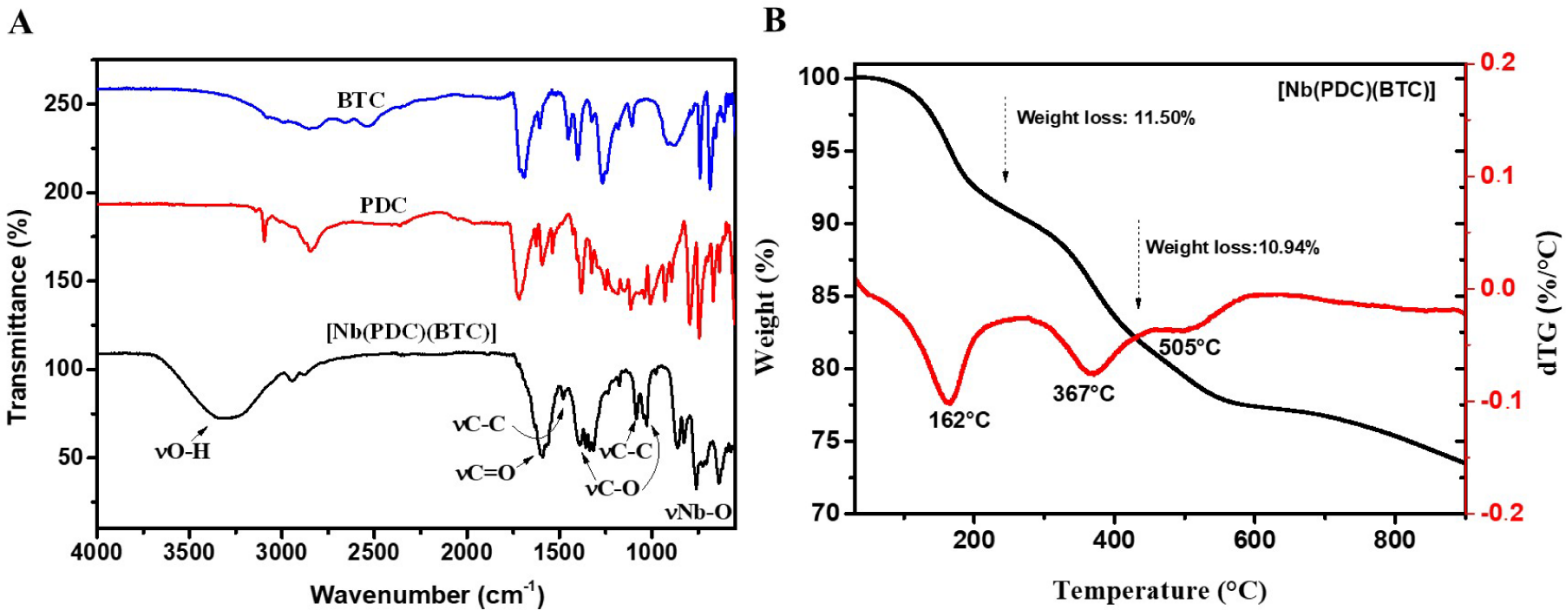
(A) FTIR spectra of 1,3,5-benzenetricarboxylic acid (BTC), 2,5-pyridinedicarboxylic acid (PDC) and [Nb­(PDC)­(BTC)]. (B) Thermogravimetry analysis curve of [Nb­(PDC)­(BTC)], showing the main thermal decomposition steps and corresponding weight losses.

Prominent absorption bands observed at 1386, 1482, and 1600 cm^–1^ correspond to the symmetric and asymmetric stretching vibrations of carboxylate groups (−COO^–^), as well as to the stretching modes of aromatic C=C bonds, further confirming the coordination of the BTC and PDC ligands to the niobium center. Additionally, the broadband centered around 3400 cm^–1^ is attributed to the presence of hydroxyl groups and/or physically adsorbed or coordinated water molecules within the structure of the synthesized material. Similar results were reported by Dutta et al. in a nickel-based MOF derived from BTC, which also exhibited characteristic carboxylate stretching vibrations and metal–oxygen bonding bands in its FTIR spectrum.^15^

The TGA analysis of the [Nb­(PDC)­(BTC)] revealed three distinct thermal events (Figure 1B). The first mass loss, approximately 11.5%, occurred at around 162 °C and can be attributed to the removal of physically adsorbed or weakly coordinated water molecules within the porous structure of the MOF. This behavior reflects the presence of channels or cavities capable of retaining water, a characteristic feature of MOF. The second thermal event, observed around 367 °C, can be attributed to the partial decomposition of the organic ligands, indicating the onset of structural degradation of the MOF. At this stage, an additional weight loss of 10.94% was recorded, which is attributed to the degradation of the BTC and PDC ligands coordinated to the metal centers. Similar thermal behavior was reported by Squizzatto et al. supporting the interpretation of this decomposition process.^9^ A third, less noticeable mass loss occurred around 505 °C, suggesting the final decomposition of residual organic components and the possible formation of metal oxides as decomposition products. The thermal stability of [Nb­(PDC)­(BTC)] up to approximately 350 °C demonstrates notable structural integrity, supporting its potential use as a catalytic support in H_2_ evolution reactions that demand thermal resistance and chemical stability under diverse operating conditions.

The XRD pattern (Figure 2A) of the compound [Nb­(PDC)­(BTC)] was compared with the reference standard (1217843, corresponding to C_40_H_100_Nb_8_O_30_, Figure 2B). The partial match between the experimental and reference diffractograms supports the successful formation of the expected crystalline phase. Additionally, XRD analysis of the [Nb­(PDC)­(BTC)]/Co/Pt-NPs catalyst was performed, as shown in Figure S1A. The diffractogram was compared with reference crystallographic patterns of niobium oxide, platinum, and cobalt, revealing good agreement between the experimental and reference patterns, which confirms the successful synthesis of the metal nanoparticles decorated on the support. Variations in peak intensities and slight shifts in 2θ values may arise from differences in particle size, preferred crystallographic orientation, or the presence of trace residual impurities. Taken together, the structural features observed in the diffractogram indicate that the material was successfully synthesized and exhibits characteristics compatible with its potential use as a catalyst in H_2_ evolution reactions.

**Figure 2:**
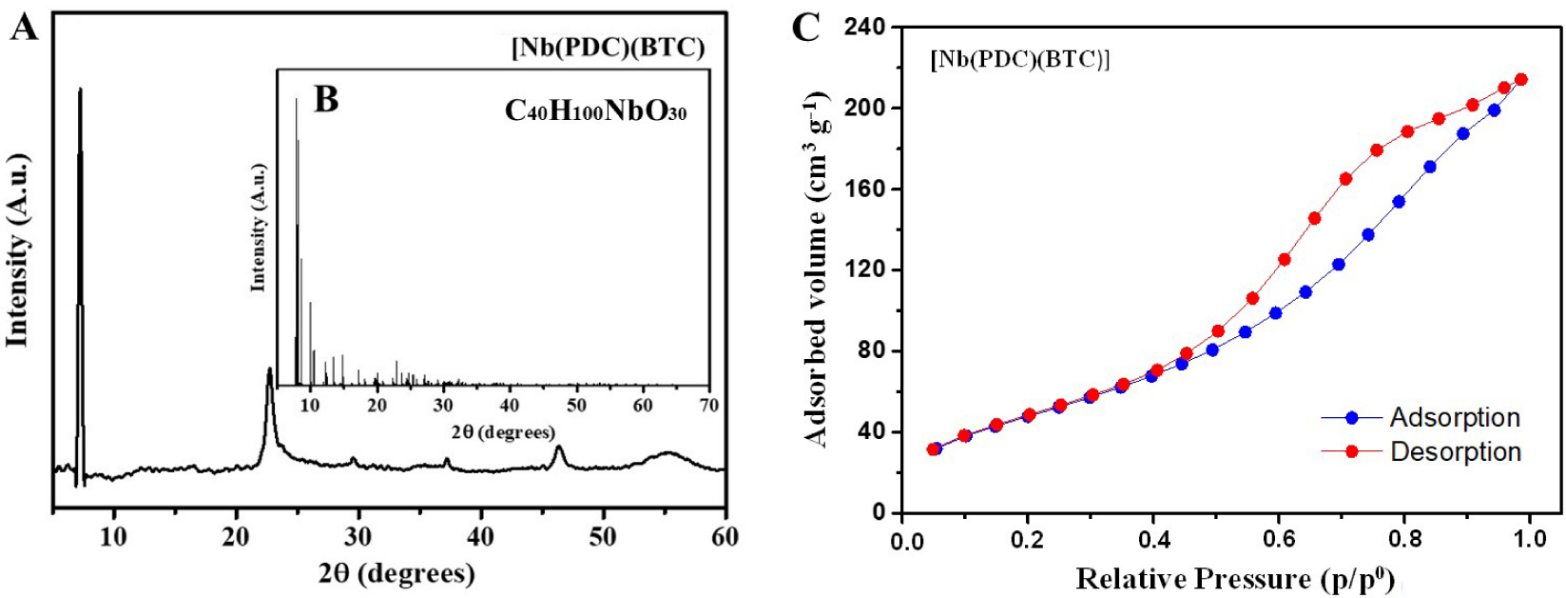
(A) X-ray diffraction pattern of [Nb­(PDC)­(BTC)]. (B) Reference crystallographic pattern (1217843), showing good agreement with the experimental data of [Nb­(PDC)­(BTC)]. (C) N_2_ adsorption–desorption isotherm curve for [Nb­(PDC)­(BTC)].

The surface area of the materials [Nb­(PDC)­(BTC)] and [Nb­(PDC)­(BTC)]/Co/Pt-NPs was measured using the BET method. The N_2_ adsorption–desorption isotherm, shown in Figure 2C, indicated that the [Nb­(PDC)­(BTC)] exhibited a type IV isotherm, typical of mesoporous structures (2–50 nm). The synthesized material had a specific surface area of 183.963 m^2^ g^–1^ and a pore volume of 0.345 cm^3^ g^–1^, demonstrating a high contact area and a well-developed porous network. A larger surface area improves the accessibility of catalytic sites to NaBH_4_ and enhances interaction with H_2_ molecules, thereby increasing H_2_ generation efficiency.^9^ These results show that the [Nb­(PDC)­(BTC)] has favorable properties for catalytic uses, especially its notable porosity and ability to function as an effective support under various reaction conditions.

The [Nb­(PDC)­(BTC)]/Co/Pt-NPs catalyst also exhibited a type IV isotherm (Figure S1B), with a specific surface area of 21.392 m^2^ g^–1^ and a pore volume of 0.0469 cm^3^ g^–1^. The reduction in surface area compared to [Nb­(PDC)­(BTC)] can be attributed to the incorporation of Co/Pt nanoparticles into the support. The presence of these nanoparticles likely leads to partial pore blocking, reducing surface accessibility and, consequently, the overall available surface area.

The morphology of the [Nb­(PDC)­(BTC)] support, examined by SEM (Figure 3A), showed a crystalline structure with rough, irregular surfaces. Elemental analysis by EDS (Figure 3B) confirmed the presence of Nb, O, and C, indicating the successful formation of the coordination compound.

**Figure 3:**
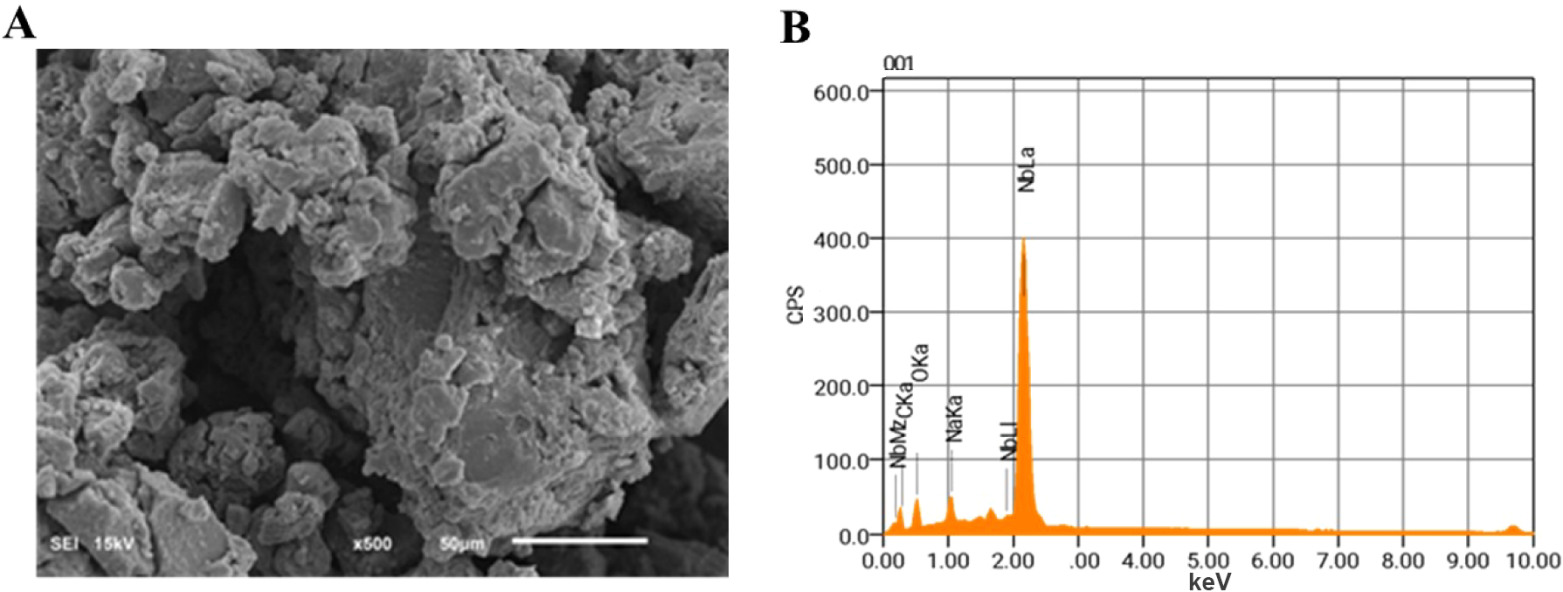
(A) Scanning Electron Microscopy (SEM) image and (B) Energy Dispersive Spectroscopy (EDS) of [Nb­(PDC)­(BTC)].

Subsequently, the [Nb­(PDC)­(BTC)]/Co/Pt-NPs catalyst was characterized by HRTEM, and the results are shown in Figure 4 and Figure S1C. The micrograph (Figure 4AI) reveals Co/Pt NPs anchored on the surface of the [Nb­(PDC)­(BTC)] support. The NPs exhibited a spherical morphology and a uniform distribution (Figure 6AII), with an average diameter of 3.7 ± 0.6 nm (Figure 4B). In addition, complementary EDS analysis (Figure 4C) confirmed the presence of Co, Pt, and Nb, further corroborating the successful functionalization of the support with metallic NPs.

**Figure 4:**
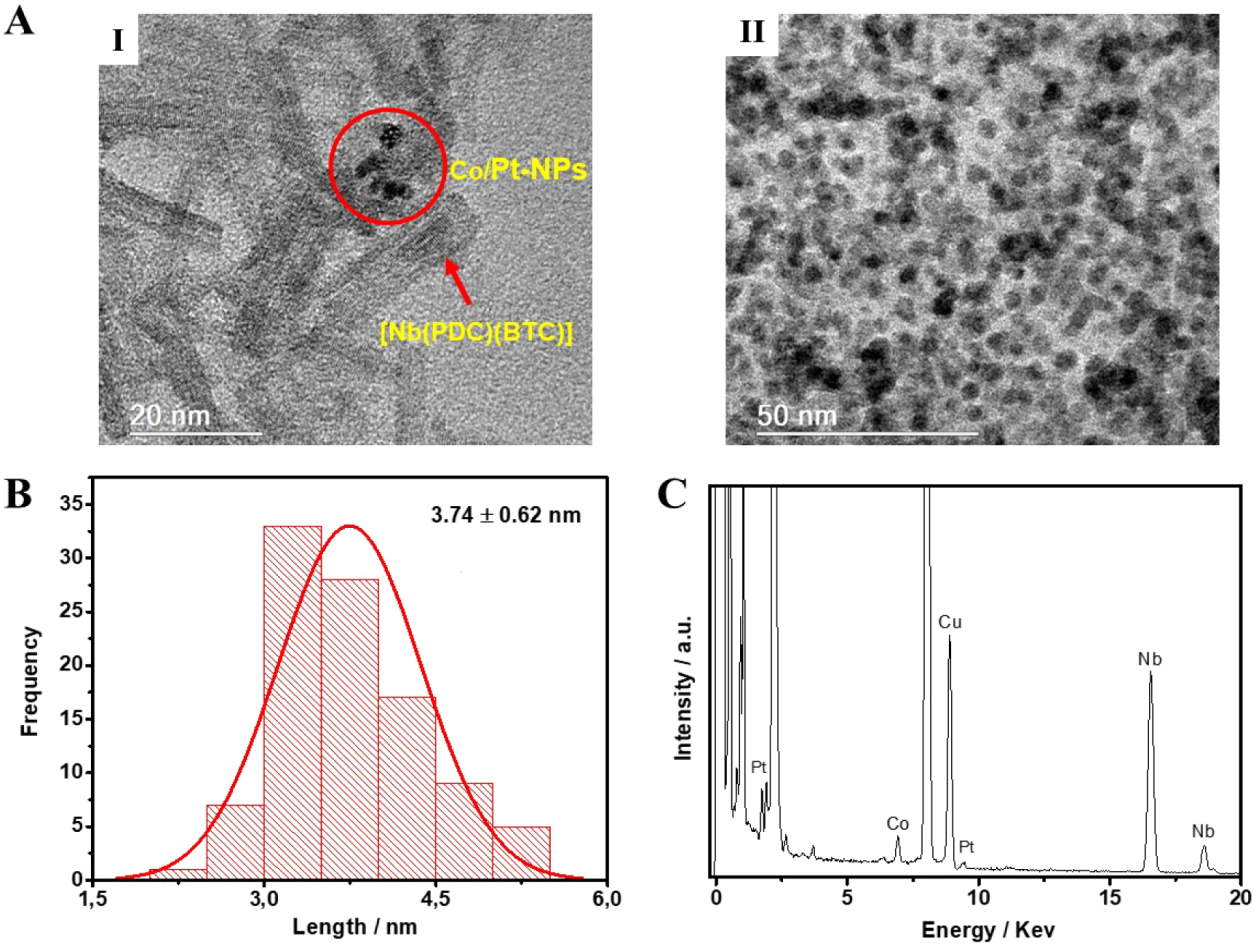
(A) High-Resolution Transmission Electron Microscopy (HRTEM) images, (B) Dynamic Light Scattering (DLS) measurements and (C) Energy Dispersive Spectroscopy (EDS) of [Nb­(PDC)­(BTC)]/Co_60_/Pt_40_-NPs catalyst.

### 3.2 Optimization of Parameters for Hydrogen Generation from NaBH_4_

#### 3.2.1 Synergistic Effect of the MOF Support and Co/Pt Bimetallic Nanoparticle

Hydrogen generation from NaBH_4_ hydrolysis can be accelerated in the presence of transition metal and noble metal-based catalysts. In this work, the catalytic performance of Co/Pt nanoparticles with different compositions, supported on [Nb­(PDC)­(BTC)], was systematically evaluated (Figure S2). The H_2_ evolution profiles show a strong dependence on the bimetallic ratio, with the Co_60_/Pt_40_ formulation exhibiting the fastest kinetics and achieving complete conversion in the shortest time (Figure S2A). To elucidate the role of the support, the Co_60_/Pt_40_ NPs were also tested in the absence of [Nb­(PDC)­(BTC)]. The unsupported catalyst exhibited significantly lower activity, with a hydrogen generation rate (HGR) of only 115 mL min^–1^ g^–1^, while the supported system reached 1964 mL min^–1^ g^–1^ (Figure S2B). This approximately 17-fold increase highlights the critical role of the MOF support in promoting nanoparticle dispersion, increasing the number of accessible active sites, and facilitating mass transfer of BH_4_^–^ and water molecules.

These results demonstrate a pronounced synergistic interaction between the [Nb­(PDC)­(BTC)] structure and the Co/Pt bimetallic nanoparticles, leading to superior catalytic efficiency. Consequently, the [Nb­(PDC)­(BTC)]/Co_60_/Pt_40_-NPs material was selected as the ideal catalyst for subsequent kinetic and mechanistic studies.

#### 3.2.2 Optimization of Catalyst Loading to Increase H_2_ Production

After establishing the ideal bimetallic composition, the effect of catalytic loading on NaBH_4_ hydrolysis was investigated in the range of 0.5 to 10.0 mmol %. The hydrogen evolution profiles (Figure S3A) show that increasing the catalyst concentration accelerates the initial reaction rate, as expected due to the higher density of accessible active sites. However, the corresponding HGR values (Figure S3B) reveal a nonlinear behavior, with the maximum specific activity obtained at the lowest loading (0.5 mmol %, 2001 mL min^–1^ g^–1^), followed by a slight decrease at 1.0 mmol % (1954 mL min^–1^ g^–1^) and a sharp drop at higher concentrations (3.0–10.0 mmol %).

This trend indicates that, although larger amounts of catalyst reduce the reaction time, the mass-normalized activity is reduced due to partial aggregation of nanoparticles, increased diffusional limitations, and inefficient use of active sites at high loadings. In contrast, at 0.5 mmol %, the catalyst is more effectively dispersed on the [Nb­(PDC)­(BTC)] support, maximizing metal exposure and facilitating reagent access, resulting in superior intrinsic activity. Therefore, 0.5 mmol % was selected as the ideal loading, providing the best balance between kinetic performance, metal utilization, and H_2_ generation efficiency.

#### 3.2.3 Effect of NaBH_4_ Concentration to Increase H_2_ Production

The effect of NaBH_4_ concentration on hydrolysis kinetics was also systematically investigated (Figure S3). At low substrate concentrations (0.50 and 0.75 mmol), similar initial rates were observed, indicating that the catalyst surface is not fully saturated and that the reaction occurs under kinetically controlled conditions (Figure S4A). However, increasing the NaBH_4_ concentration to 1.0 and 1.5 mmol led to a progressive decrease in the H_2_/NaBH_4_ ratio and a lower apparent conversion over the same reaction time. This behavior suggests that excess borohydride does not translate into higher H_2_ productivity under the studied conditions. The inhibitory effect is consistent with the accumulation of sodium metaborate (NaBO_2_), which can precipitate on the catalyst surface and block the active sites, thus limiting BH_4_^–^ adsorption and subsequent hydrolysis steps.^16^ Furthermore, higher concentrations of NaBH_4_ increase the viscosity of the reaction medium, which hinders the mass transfer of BH_4_^–^ and B­(OH)_4_^–^ species toward and away from the active sites.^8,17^ As a result, the reaction becomes partially diffusion-limited, reducing the effective conversion rate.

Kinetic analysis (Figure S4B) revealed a negative fractional reaction order with respect to NaBH_4_ (−0.60), confirming that excess substrate exerts an inhibitory effect on the catalytic process. Such behavior is frequently reported in heterogeneous hydrolysis systems with NaBH_4_ and is typically associated with surface saturation, competitive adsorption of reaction intermediates/byproducts, and blocking of catalytically active metal sites.^9,16^ These results indicate that optimal H_2_ generation is achieved at moderate NaBH_4_ concentrations, where a balance is maintained between substrate availability, byproduct accumulation, and mass transfer limitations.

#### 3.2.4 Effect of NaOH Concentration to Increase H_2_ Production from NaBH_4_ Hydrolysis

The effect of NaOH concentration on NaBH_4_ hydrolysis was evaluated using the catalyst [Nb­(PDC)­(BTC)]/Co_60_/Pt_40_-NPs (Figure S5). In the absence of NaOH, the reaction proceeds significantly more slowly, reaching a lower H_2_/NaBH_4_ ratio and a hydrogen HGR of 1960 mL min^–1^ g^–1^, indicating limited borohydride stability and less favorable catalytic conditions. With the addition of NaOH (0.01–0.20 mol L^–1^), a marked acceleration of the reaction kinetics is observed, with steeper initial slopes in the H_2_ evolution curves (Figure S5A). HGR increases to 2806, 3159, and 3214 mL min^–1^ g^–1^ for concentrations of 0.01, 0.05, and 0.10 mol L^–1^, respectively, followed by a slight decrease at 0.20 mol L^–1^ (3156 mL min^–1^ g^–1^) (Figure S5B). These results indicate an optimal alkaline concentration around 0.10 mol L^–1^. The increase in catalytic performance under alkaline conditions can be attributed to multiple synergistic effects. First, NaOH stabilizes NaBH_4_ in solution, suppressing its noncatalytic hydrolysis and ensuring that H_2_ evolution occurs predominantly on the catalyst surface.^8,18^ Second, OH^–^ species participate in the gradual hydrolysis pathway, facilitating nucleophilic attack on adsorbed BH_4_^–^, promoting the formation of B–OH intermediates on the surface and the subsequent release of H_2_, ultimately resulting in B­(OH)_4_^–^. Third, hydroxide adsorption on the Co–Pt surface can increase the electron density of metal sites, improving BH_4_^–^ activation and H–H recombination kinetics.^19,20^

The approximately 64% increase in the HGR generation rate in alkaline medium, compared to the system without NaOH, highlights the crucial role of pH in modulating both substrate stability and interfacial catalytic chemistry. However, at higher NaOH concentrations (0.20 mol L^–1^), the slight decline in activity suggests the onset of mass transfer limitations and/or competitive adsorption of excess OH^–^ at the active sites, which may hinder BH_4_^–^ access to the catalytic surface. This volcano-like behavior reflects a balance between borohydride stabilization, surface activation, and diffusion effects.

In general, these results demonstrate that the alkalinity of the reaction medium is a fundamental kinetic parameter governing the hydrolysis of NaBH_4_ on Co–Pt bimetallic catalysts supported on MOFs, and that an optimal OH^–^ concentration maximizes H_2_ generation by simultaneously increasing substrate stability, surface reactivity, and interfacial charge transfer, avoiding site blockage or transport limitations.

#### 3.2.5 Effect of Temperature to Increase H_2_ Production from NaBH_4_ Hydrolysisi Using [Nb­(PDC)­(BTC)]/Co_60_/Pt_40_-NPs

Temperature plays a key role in many catalytic reactions, as higher temperatures increase molecular collisions and enhance reaction kinetics.^9^ This was evident in the hydrolysis of NaBH_4_ by the catalyst [Nb­(PDC)­(BTC)]/Co_60_/Pt_40_-NPs (Figure 5A). Increasing the temperature from 288.15 to 328.15 K significantly accelerated the evolution of H_2_, as evidenced by the steeper initial slopes and the shorter time required to reach the theoretical stoichiometric H_2_/NaBH_4_ ratio. At 288.15 K, the reaction proceeded slowly, indicating that the process is kinetically limited under these conditions. In contrast, at temperatures ≥308.15 K, a rapid release of hydrogen was observed in the first few minutes, suggesting enhanced surface reaction rates and better mass transport of the BH_4_^–^/B­(OH)_4_^–^ species to and from the catalytic interface.

**Figure 5:**
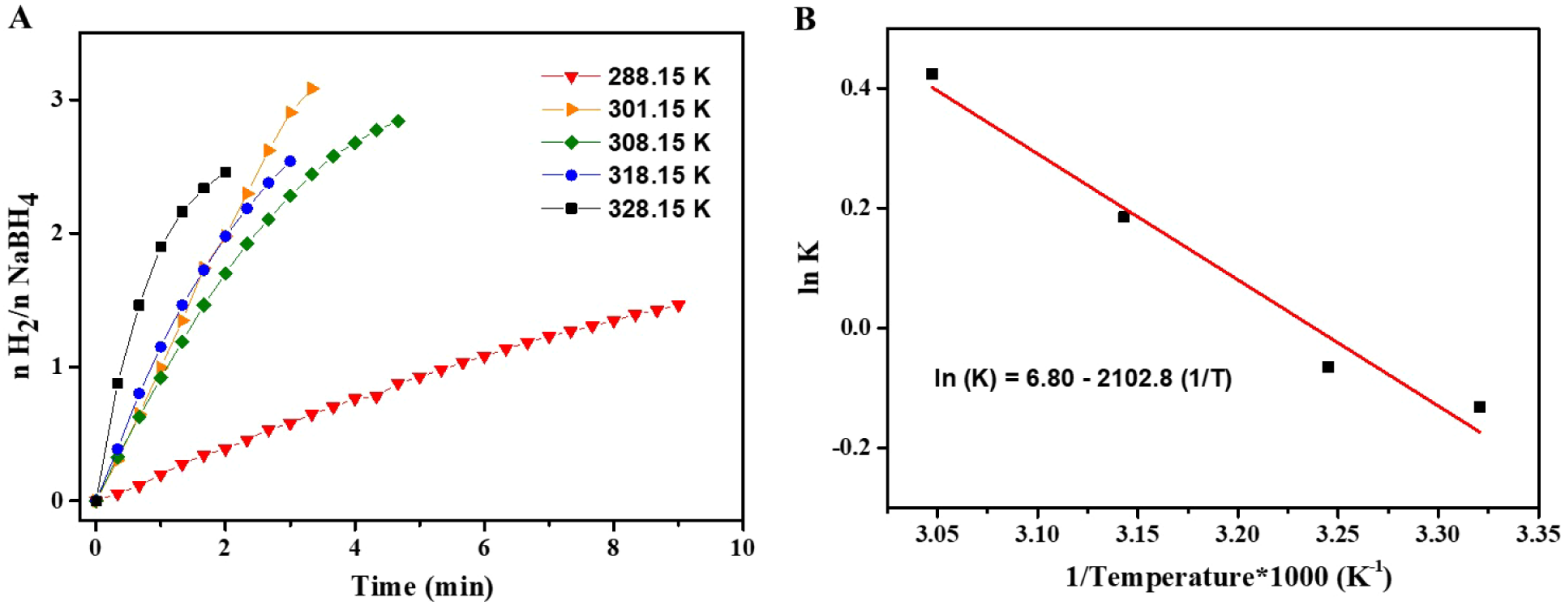
(A) Effect of temperature on H_2_ evolution catalyzed by [Nb­(PDC)­(BTC)]/Co_60_/Pt_40_-NPs. (B) Arrhenius plot (ln k vs 1/T) used to determine the apparent activation energy. Reaction conditions: 0.5 mmol of Co_60_/Pt_40_ NPs supported on 10 mg of [Nb­(PDC)­(BTC)], 1.00 mL of NaBH_4_ solution (0.500 mol L^–1^) in NaOH (0.100 mol L^–1^).

The temperature dependence reflects an increase in the intrinsic rate constant (k), consistent with Arrhenius-type behavior. The linear relationship obtained in the Arrhenius plot (Figure 5B) indicates that the reaction is governed by a single rate-determining step dominant in the investigated temperature range, with negligible diffusional constraints. From the slope (−E_a_/R), an apparent activation energy of ∼17.4 kJ mol^–1^ was calculated, which is considerably lower than the values reported for many heterogeneous Co- and Pt-based systems. The data were fitted to a linear equation model with a correlation coefficient of 0.974. This low E_a_ value suggests that the synergistic interaction between the active sites of Co and Pt, along with the electronic modulation provided by the [Nb­(PDC)­(BTC)] support, facilitates BH_4_^–^ activation and H–H recombination.

Furthermore, the significant increase in reaction rate at higher temperatures indicates that the catalytic surface remains stable and active, without significant deactivation, corroborating the structural robustness of the MOF-supported bimetallic nanoparticles under alkaline hydrolytic conditions. Overall, these results confirm that temperature primarily affects the reaction kinetics at the surface, rather than inducing mass transfer restrictions, reinforcing the high catalytic efficiency of the [Nb­(PDC)­(BTC)]/Co_60_/Pt_40_ system for NaBH_4_ hydrolysis. These findings are consistent with previous studies that investigated the effect of temperature on H_2_ production during NaBH_4_ hydrolysis, demonstrating that higher temperatures lead to enhanced reaction kinetics.^8,21^

This finding highlights the superior performance of the proposed catalyst compared to other Co and Pt-based catalytic systems, as summarized in Table 1.

**Table 1:** Comparison of Different Catalysts for Hydrogen Production from Sodium Borohydride (NaBH_4_) Hydrolysis^a^

Catalyst	HGR (mL min^–1^ g_cat_^–1^)	E_a_ (kJ mol^–1^)	Temperature (K)	Reference
Pt/Co(OH)_2_-B	9315	47.3	303.15	^22^
CNSs@Pt_0.1_Co_0.9_	8943	38.0	NS	^23^
Pt_0.25_/Co	1511	NS	NS	^24^
CoPt-PEDOT:PSS/MWCNT	6900	47.3	298.15	^25^
Pt/Co_3_O_4_	4713	43.5	298.15	^26^
[Nb(PDC)(BTC)]/Co_60_/Pt_40_-NPs	3214	17.4	301.15	This work

a CoPt-PEDOT:PSS/MWCNT: CoPt nanoparticles supported on poly­(3,4-ethylenedioxythiophene)/poly­(styrenesulfonate) (PEDOT:PSS) functionalized multiwalled carbon nanotubes (MWCNTs); CNSs@Pt_0.1_Co_0.9_: carbon nanospheres supporting ultrafine bimetallic Pt–Co nanoparticles; E_a_: activation energy; HGR: hydrogen generation rate; NS: not specified; Pt/Co­(OH)_2_-B: Pt single-atomic-site loaded on borate anion-intercalated Co­(OH)_2_ nanoflakes; Pt_0.25_/Co: subnanometric Pt clusters supported Co aerogel; Pt/Co_3_O_4_: Pt–Pd nanoparticles loaded on Co_3_O_4_ support material.

#### 3.2.6 Mechanistic Aspects of H_2_ Generation via NaBH_4_ Hydrolysisi Using [Nb­(PDC)­(BTC)]/Co_60_/Pt_40_-NPs

The catalytic hydrolysis of NaBH_4_ using the [Nb­(PDC)­(BTC)]/Co_60_/Pt_40_ nanocatalyst follows a multistep surface-mediated mechanism,^27^ characterized by a synergistic interplay between the bimetallic active sites and the [Nb­(PDC)­(BTC)] support.

The process initiates with the competitive adsorption of BH_4_^–^ and water molecules onto the surface of the Co_60_/Pt_40_ nanoparticles (Figure 6). In this bimetallic system, the electronic structure is modified by the “ligand effect” and “strain effect,” where the electron transfer between Co and Pt optimizes the *d*-band center, enhancing the binding energy of the reactants while preventing the overly strong adsorption of the BO_2_^–^ byproduct.^27^ As the BH_4_^–^ species anchors to the metal surface, the rate-limiting step involves the stepwise cleavage of the B–H bonds. The oxophilic nature of Co facilitates the dissociation of water molecules into hydroxyl groups OH^–^ and protons H^+^, or adsorbed hydrogen atoms (H_ads_). Simultaneously, the Pt sites, which possess a lower activation energy for hydrogen recombination, act as the primary centers for the formation of molecular hydrogen (H_2_). Each hydride removed from the boron atom is replaced by a hydroxyl group from the activated water, progressively transforming the borohydride into a tetrahydroxyborate intermediate—B­(OH)_4_^–^—and, ultimately, into sodium metaborate (NaBO_2_) (eq 2).^27^

**Figure 6:**
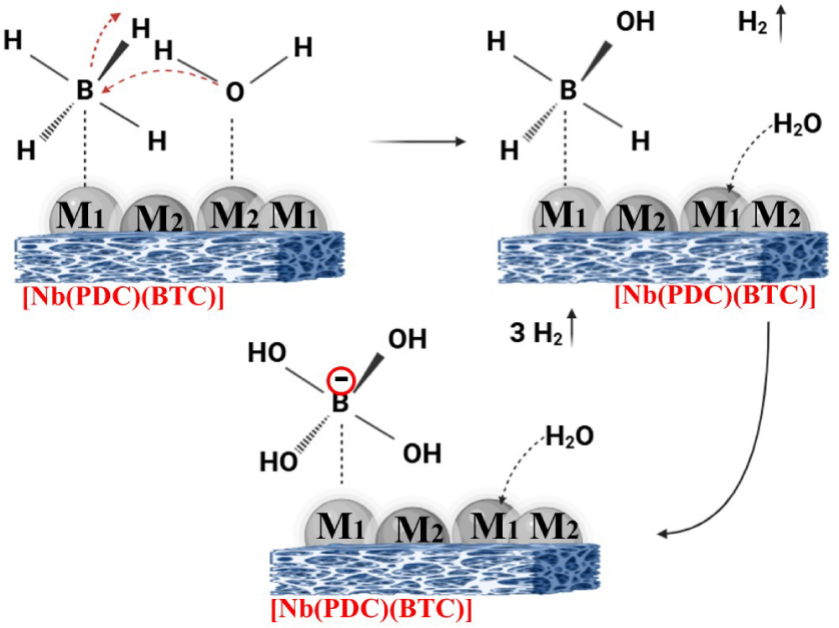
Proposed catalytic mechanism for NaBH_4_ hydrolysis over the bimetallic [Nb­(PDC)­(BTC)]/Co_60_/Pt_40_ nanoparticles. BH_4_^–^ adsorption occurs at the Co–Pt active interface (M_1_ = Co, M_2_ = Pt), followed by stepwise activation of B–H bonds and formation of surface hydride species. Water-assisted proton transfer leads to H_2_ evolution, while hydroxyl groups progressively substitute hydride ligands to yield B­(OH)_4_^–^ as the intermediate product. The Nb-based MOF support promotes nanoparticle dispersion and interfacial charge transfer, enhancing catalytic turnover and facilitating desorption of H_2_ and borate species.

The [Nb­(PDC)­(BTC)] support plays a crucial structural and electronic role beyond mere stabilization.^28^ The high surface area and porous architecture of the MOF ensure a high dispersion of the Co_60_/Pt_40_ nanoparticles, preventing sintering and maintaining a high density of active sites.^28^ Furthermore, the electron-withdrawing or donating characteristics of the Nb nodes can modulate the electron density of the supported nanoparticles, a phenomenon known as Strong Metal–Support Interaction (SMSI). This interaction further accelerates the H–H recombination and the subsequent desorption of H_2_ gas. The final stage of the mechanism involves the desorption of the NaBO_2_ byproduct into the aqueous medium, which vacates the catalytic sites for a new reaction cycle, resulting in a theoretical yield of 4 mol of H_2_ per mole of NaBH_4_ (eq 2).^28^

2 $${\mathrm{NaBH}}_{4(\mathrm{aq})}{+2H}_{2}O_{\left(l\right)}\overset{\left[\left(Nb\left(PDC\right)\left(BTC\right)/Co_{60}/Pt_{40}\right)\right]}{\to }{\mathrm{NaBO}}_{2(\mathrm{aq})}{+4H}_{2(g)}$$

## 4 Conclusions

This study demonstrates the successful synthesis and application of an Nb-based MOF functionalized with bimetallic Co/Pt nanoparticles for H_2_ generation via NaBH_4_ hydrolysis. The catalyst [Nb­(PDC)­(BTC)]/Co_60_/Pt_40_-NPs was characterized by multiple analytical techniques, including FTIR, TGA, XRD, BET, SEM, and TEM. The material exhibited preserved structural integrity and a high specific surface area (183.963 m^2^ g^–1^), an essential feature for its performance as a catalytic support. Notably, the use of Nb, a strategic element for which Brazil holds some of the world’s largest reserves, underscores the importance of developing locally abundant, technologically valuable materials. The optimized catalyst achieved an HGR of 3214 mL min^–1^ g^–1^ and E_a_ of 17.45 kJ mol^–1^, evidencing excellent activity and favorable kinetics. Overall, these results highlight the potential of Nb-based MOFs to advance sustainable H_2_ production and contribute to the development of strategic energy materials.

## Supplementary Material



## Notes

Supplementary characterization data for the [Nb­(PDC)­(BTC)]/Co_60_/Pt_40_ NPs and optimization studies evaluating the effects of Co/Pt composition, nanoparticle loading, NaBH_4_ and NaOH concentrations, and [Nb­(PDC)­(BTC)] support on H_2_ evolution via NaBH_4_ hydrolysis and hydrogen generation rate (HGR) (PDF)

## References

[1] Lee H., Lee J., Kang S. W., Kim D., Kim I., Koo Y. (2024). Effects of
sector coupling on the decarbonization potential of the manufacturing
sector–an integration of the power, hydrogen, and manufacturing
sectors. Energy Strat. Rev..

[2] Meda U. S., Rajyaguru Y. V., Pandey A. (2023). Generation of green
hydrogen using
self-sustained regenerative fuel cells: Opportunities and challenges. Int. J. Hydrogen Energy.

[3] Maka A. O. M., Mehmood M. (2024). Green hydrogen energy
production: Current status and
potential. Clean Energy.

[4] Abdalla A. M., Hossain S., Nisfindy O. B., Azad A. T., Dawood M., Azad A. K. (2018). Hydrogen production,
storage, transportation and key
challenges with applications: A review. Energy
Convers. Manage..

[5] Sperandio G. H., de Carvalho J. P., de Jesus C. B. R., Junior I. M., de Oliveira K. L. A., Puiatti G. A. (2024). Hydrogen evolution from NaBH4 using novel
Ni/Pt nanoparticles decorated on a niobium-based composite. Int. J. Hydrogen Energy.

[6] Feng Y., Yang H., Wang X., Hu C., Jing H., Cheng J. (2022). Role of transition metals in catalyst
designs for oxygen evolution
reaction: A comprehensive review. Int. J. Hydrogen
Energy.

[7] Kang N., Djeda R., Wang Q., Fu F., Ruiz J., Pozzo J. (2019). Efficient “Click”-Dendrimer-Supported
Synergistic Bimetallic Nanocatalysis for Hydrogen Evolution by Sodium
Borohydride Hydrolysis. ChemCatChem.

[8] Souza E. I. P., Favero U. G., Sperandio G. H., Andrade T. A., Moreira R. P. L., Hespanhol M. C. (2025). Sustainable
nanocatalyst synthesized from battery waste
for enhanced hydrogen evolution: A circular economy approach. J. Environ. Chem. Eng..

[9] Squizzatto E. P., Andrade T. D. A., Lopes Moreira R. P., Guimarães L. D. M., da Silva M. J., Novaes F. J. M., de Jesus J. R. (2024). Development of a
Niobium-Based Coordination Compound with Catalytic Applications for
Green Hydrogen Evolution. Processes.

[10] Jiang S., Xue D., Zhang J. (2022). Optimizing Atomically Dispersed Metal Electrocatalysts
for Hydrogen Evolution: Chemical Coordination Effect and Electronic
Metal Support Interaction. Chem. - Asian J..

[11] Coelho L. O., Sperandio G. H., Chagas da Silva R., Lopes Moreira R. P., de Jesus J. R. (2024). Niobium Metal–Organic
Framework Is an Efficient
Catalytic Support for the Green Hydrogen Evolution Process from Metal
Hydride. Processes.

[12] Alves A. R., Coutinho A. D. R. (2015). The The Evolution of the Niobium Production in Brazil. Mat. Res..

[13] de
Jesus J. R., Ribeiro I. S., de Carvalho J. P., de Oliveira K. L. A., Moreira R. P. L., da Silva R. C. (2024). Successful synthesis
of eco-friendly Metal-Organic framework ([Ni­(BDC)]­n) allows efficient
extraction of multiresidues pesticides and dyes from fish samples. Microchem. J..

[14] Gómez C., Rodríguez-Páez J. (2018). The effect
of the synthesis conditions
on structure and photocatalytic activity of Nb2O5 nanostructures. Process. Appl. Ceram..

[15] Dutta M., Bora J., Chetia T., Sarmah K., Guha A. K., Chetia B. (2025). Fabrication of CuFe2O4 decorated Ni-BTC MOF for boosting
the reduction of nitroaromatic pollutants. J.
Mol. Struct..

[16] Chou C.-C., Hsieh C.-H., Chen B.-H. (2015). Hydrogen generation from catalytic
hydrolysis of sodium borohydride using bimetallic Nie–Co nanoparticles
on reduced graphene oxide as catalysts. Energy.

[17] Baydaroglu F. O., Özdemir E., Gürek A. G. (2022). Polypyrrole supported Co–W–B
nanoparticles as an efficient catalyst for improved hydrogen generation
from hydrolysis of sodium borohydride. Int.
J. Hydrogen Energy.

[18] Didehban A., Zabihi M., Babajani N. (2020). Preparation
of the efficient nano-bimetallic
cobalt-nickel catalysts supported on the various magnetic substrates
for hydrogen generation from hydrolysis of sodium borohydride in alkaline
solutions. Polyhedron.

[19] Doherty S., Knight J. G., Alharbi H. Y., Paterson R., Wills C., Dixon C. (2022). Efficient Hydrolytic Hydrogen Evolution from Sodium
Borohydride Catalyzed by Polymer Immobilized Ionic Liquid-Stabilized
Platinum Nanoparticles. ChemCatChem.

[20] Bousada G. M., da Silva V. N., de Souza B. F., de Oliveira R. S., Junior I. M., da Cunha C. H. F. (2024). Niobic acid as a support
for microheterogeneous nanocatalysis of sodium borohydride hydrolysis
under mild conditions. RSC Adv..

[21] Santos J. L. D., Machado I., Sperandio G. H., Dias G. D. C., Dias A., Lima G. M. D., Moreira R. P. (2025). Platinum
and other Metals Doped-Manganese
Tungstate as a Novel Catalytic Support for NaBH4 Hydrolysis and Hydrogen
Evolution Under Mild Conditions. J. Braz. Chem.
Soc..

[22] Guo Q., Wang X., Yang F., Zhou S., Wei K., Zhang Y. (2025). Pt single-atomic-site loaded on borate anion-intercalated
Co­(OH)­2 nanoflakes for enhanced hydrogen generation dynamics of NaBH4
hydrolysis. Chem. Eng. J..

[23] Zhang H., Zhang L., Rodríguez-Pérez I. A., Miao W., Chen K., Wang W. (2021). Carbon
nanospheres supported bimetallic Pt-Co as an efficient catalyst for
NaBH4 hydrolysis. Appl. Surf. Sci..

[24] Wang T., Zhang D., Fei J., Yu W., Zhu J., Zhang Y. (2024). Sub-nanometric Pt clusters
supported Co aerogel electrocatalyst
with hierarchical micro/nano-porous structure for hydrogen evolution
reaction. Appl. Catal., B.

[25] Wang X., Zhao Y., Peng X., Wang J., Jing C., Tian J. (2015). Synthesis and characterizations of
CoPt nanoparticles supported on
poly­(3,4-ethylenedioxythiophene)/poly­(styrenesulfonate) functionalized
multi-walled carbon nanotubes with superior activity for NaBH4 hydrolysis. Mater. Sci. Eng.: B.

[26] Bozkurt G., Özer A., Yurtcan A. B. (2019). Development of effective
catalysts
for hydrogen generation from sodium borohydride: Ru, Pt, Pd nanoparticles
supported on Co3O4. Energy.

[27] Demirci U. B., Miele P. (2014). Reaction mechanisms of the hydrolysis
of sodium borohydride: A discussion
focusing on cobalt-based catalysts. C. R. Chim..

[28] Rossin A., Tuci G., Luconi L., Giambastiani G. (2017). Metal–Organic
Frameworks as Heterogeneous Catalysts in Hydrogen Production from
Lightweight Inorganic Hydrides. ACS Catal..

