# Supplementary material for: Advanced Nb-Based MOF-Supported Co/Pt Nanocatalyst for Sustainable Hydrogen Production from Sodium Borohydride
Source: ACS Omega. 2026 May 18;11(21):31484–92. doi: 10.1021/acsomega.6c01980 (PMC13234875; doi:10.1021/acsomega.6c01980)
Supplement: Supplementary file 1 [file ao6c01980_si_001.pdf]

Supplementary Material

Advanced Nb-Based MOF-Supported Co/Pt Nanocatalyst for Sustainable Hydrogen Production from Sodium Borohydride

Giovana Barros Magalhães[a,b], Tatianny de Araujo Andrade[b], Renata Lopes Moreira[b], Renê Chagas da Silva[c], Gilberto Rodrigues da Silva Junior[c], Jemmyson Romário de Jesus[a,b,d*]

[a] Research Laboratory in bionanomaterials, LPbio, Department of Chemistry, Federal University of Viçosa, 36570-900 Viçosa, Minas Gerais, Brazil.

[b] Department of Chemistry, Federal University of Viçosa, 36570-900 Viçosa, Minas Gerais, Brazil.

[c] Department of Physics, Federal University of Viçosa, 36570-900 Viçosa, Minas Gerais, Brazil

[d] National Institute of Science and Technology of Bioanalytics-Lauro Kubota (INCTBio-LK), Instituto de Química, Universidade Estadual de Campinas, Campinas, SP 13083970, Brazil

*Corresponding author:

Prof. Jemmyson R. de Jesus

E-mail address: jemmyson.jesus@ufv.br

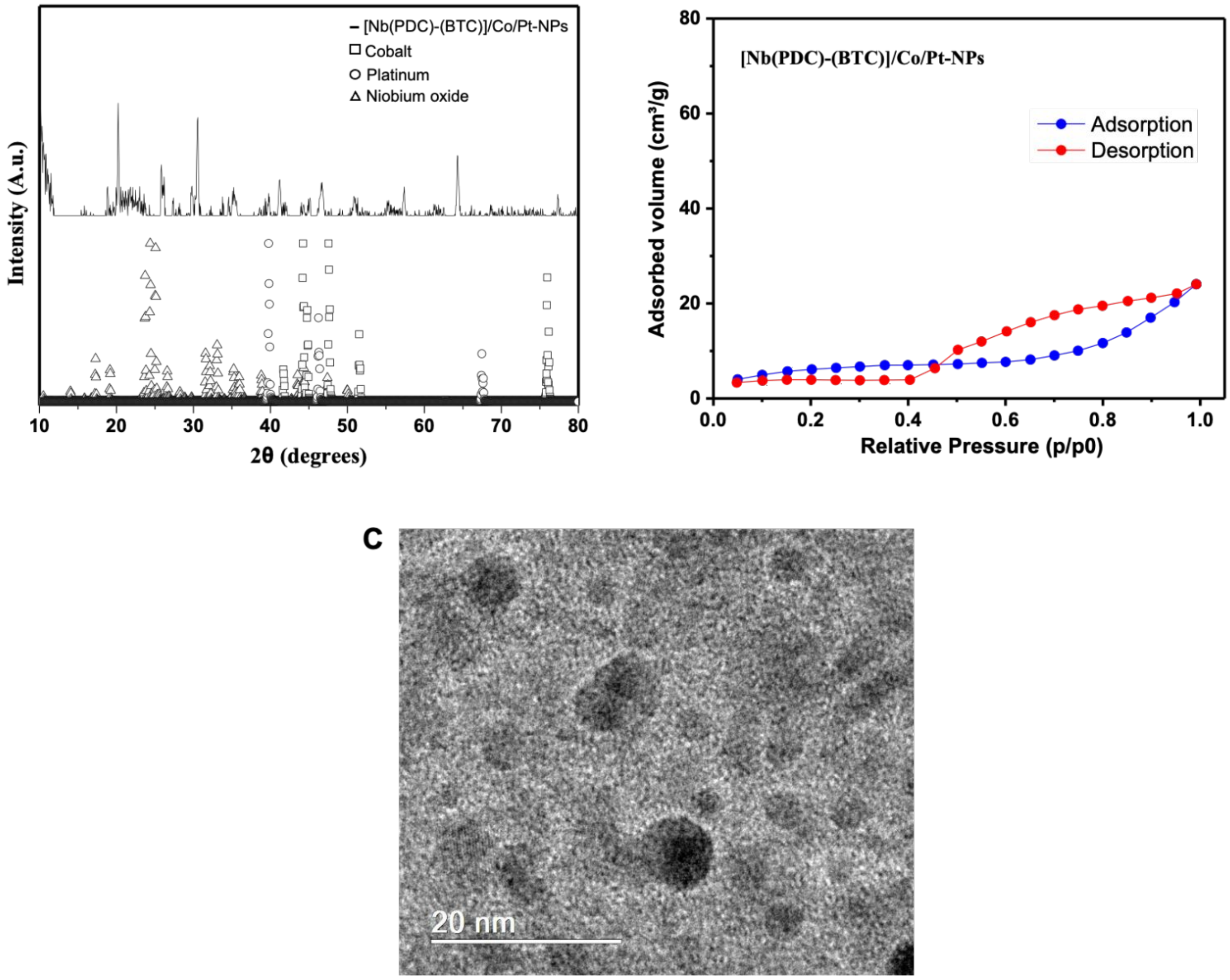

Figure S1. **(A)** X-ray diffraction pattern of [Nb(PDC)(BTC)]/CoPt-NPs, compared with reference crystallographic patterns (PDF 30871 - niobium oxide; 4802 - platinum; 5727 and 15806 - cobalt). **(B)** N_2 adsorption-desorption isotherm curve for [Nb(PDC)(BTC)]/Co/Pt-NPs. **(C)** High-Resolution Transmission Electron Microscopy (HRTEM) image of [Nb(PDC)(BTC)]/Co/Pt-NPs catalyst.

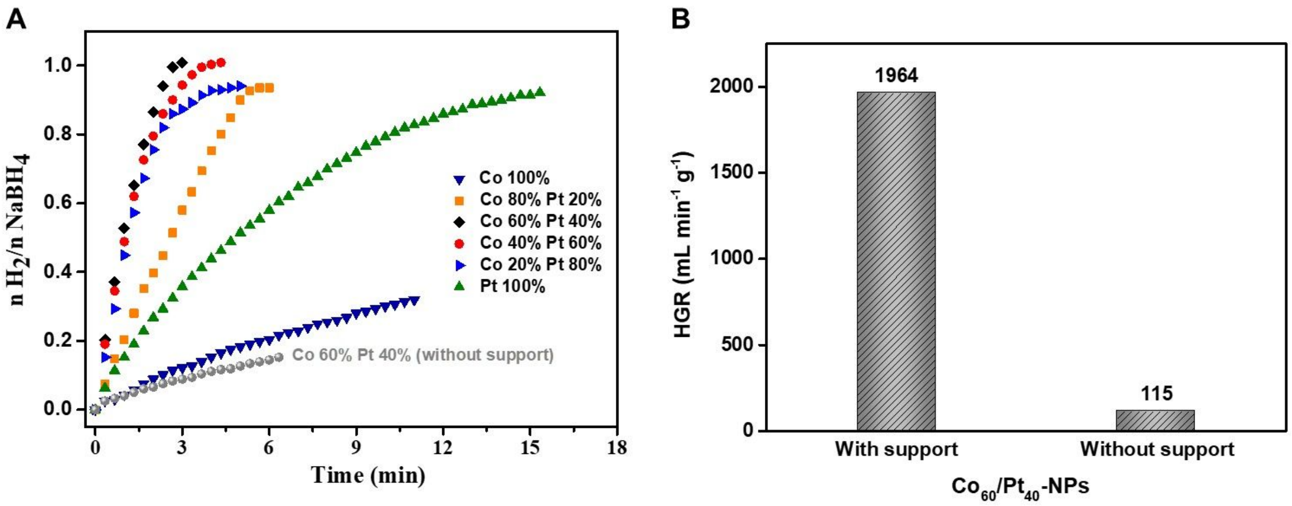

Figure S2. Effect of Co/Pt nanoparticle composition and the [Nb(PDC)(BTC)] support on **(A)** H_2 evolution from $NaBH_4$ hydrolysis and **(B)** the hydrogen generation rate (HGR). Reaction conditions: 10 mg of catalyst, 1.00 mL of 0.500 mol L^{-1} $NaBH_4$ solution, at 301.15 K.

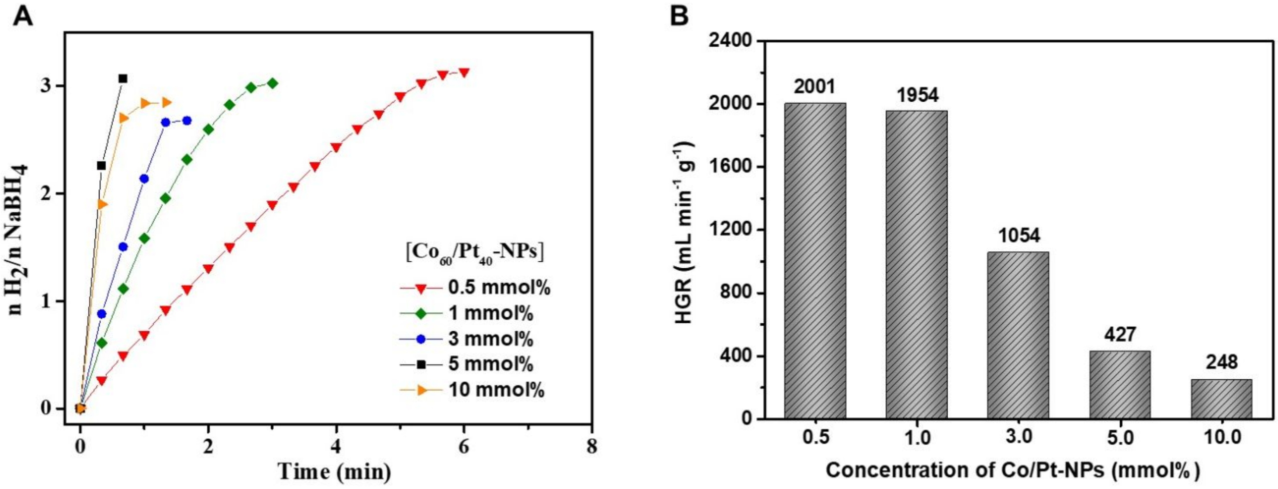

Figure S3. Effect of Co/Pt nanoparticle loading on **(A)** H_2 evolution from $NaBH_4$ hydrolysis and **(B)** the hydrogen generation rate (HGR). Reaction conditions: 10 mg of catalyst support, 1.00 mL of 0.500 mol L^{-1} $NaBH_4$ solution, at 301.15 K.

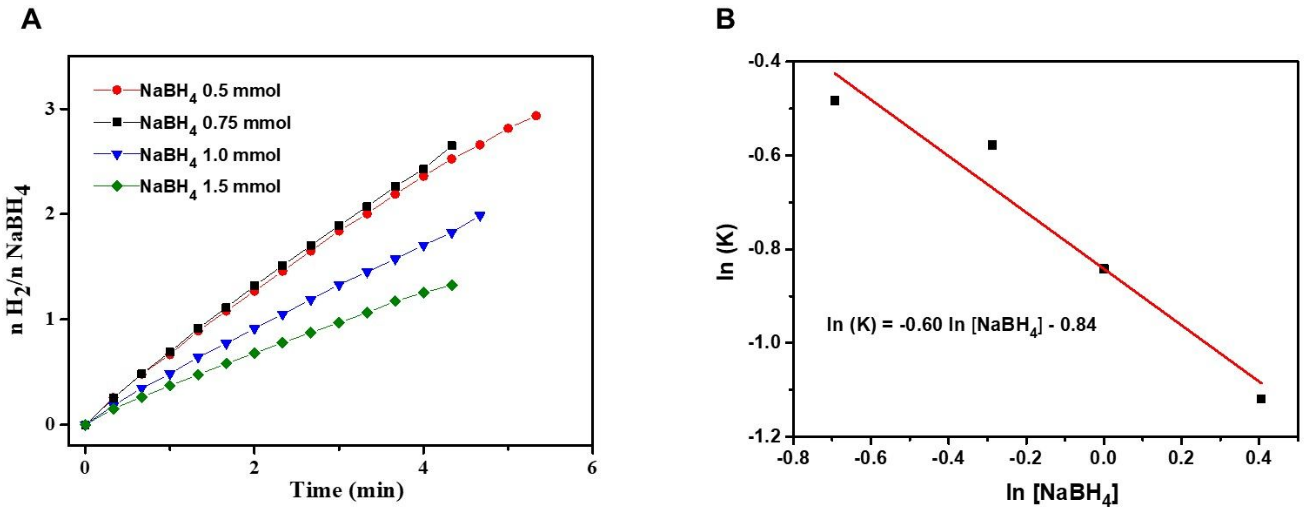

Figure S4. (A) H_2 evolution profiles at different $NaBH_4$ concentrations. **(B)** Plot of ln k versus ln [$NaBH_4$] used to determine the reaction order with respect to $NaBH_4$. Reaction conditions: Co_{60}/Pt_{40} NPs (0.5 mmol) supported on 10 mg of [Nb(PDC)(BTC)], 1.00 mL of $NaBH_4$ solution, at 301.15 K.

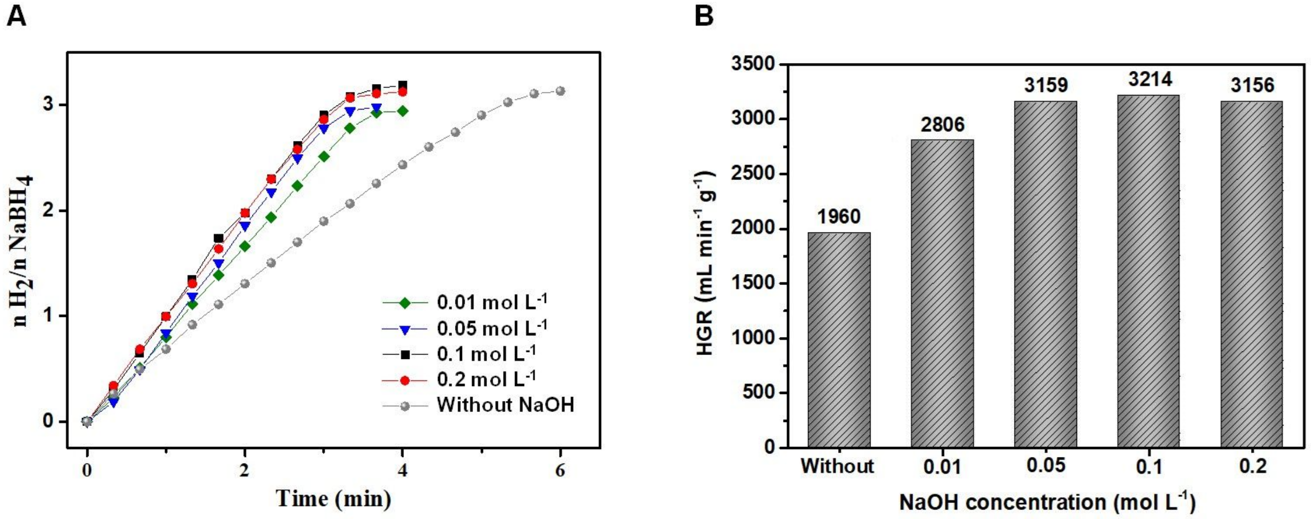

Figure S5. Effect of NaOH concentration (0.01 to 0.20 mol L^{-1}) on **(A)** H_2 evolution from $NaBH_4$ hydrolysis and **(B)** the hydrogen generation rate (HGR). Reaction conditions: Co_{60}/Pt_{40} NPs (0.5 mmol) supported on 10 mg of [Nb(PDC)(BTC)], 1.00 mL of 0.500 mol L^{-1} $NaBH_4$ solution, at 301.15 K.
